# Association Between Sexual Orientation, Mistreatment, and Burnout Among US Medical Students

**DOI:** 10.1001/jamanetworkopen.2020.36136

**Published:** 2021-02-02

**Authors:** Elizabeth A. Samuels, Dowin H. Boatright, Ambrose H. Wong, Laura D. Cramer, Mayur M. Desai, Michael T. Solotke, Darin Latimore, Cary P. Gross

**Affiliations:** 1 Department of Emergency Medicine, Alpert Medical School of Brown University, Providence, Rhode Island; 2 Department of Emergency Medicine, Yale School of Medicine, New Haven, Connecticut; 3 National Clinician Scholars Program, Yale School of Medicine, New Haven, Connecticut; 4 Department of Chronic Disease Epidemiology, Yale School of Public Health, New Haven, Connecticut; 5 Yale School of Medicine, New Haven, Connecticut; 6 Section of General Internal Medicine, Yale School of Medicine, New Haven, Connecticut

## Abstract

**Importance:**

Medical trainee burnout is associated with poor quality care and attrition. Medical students in sexual minority groups report fear of discrimination and increased mistreatment, but the association between sexual orientation, burnout, and mistreatment is unknown.

**Objective:**

To evaluate whether medical student burnout differs by sexual orientation and whether this association is mediated by experiences of mistreatment.

**Design, Setting, and Participants:**

This cross-sectional study surveyed US medical students graduating from Association of American Medical Colleges (AAMC)–accredited US allopathic medical schools who responded to the AAMC graduation questionnaire in 2016 and 2017. Statistical analyses were performed from March 15, 2019, to July 2, 2020, and from November 20 to December 9, 2020.

**Main Outcomes and Measures:**

Burnout was measured using the Oldenburg Burnout Inventory for Medical Students, and sexual orientation was categorized as either heterosexual or lesbian, gay, or bisexual (LGB). Logistic regression models were constructed to evaluate the association between sexual orientation and experiencing burnout (defined as being in the top quartile of exhaustion and disengagement burnout dimensions) and to test the mediating association of mistreatment.

**Results:**

From 2016 to 2017, 30 651 students completed the AAMC Graduation Questionnaire, and 26 123 responses were analyzed. Most respondents were younger than 30 years (82.9%) and White (60.3%). A total of 13 470 respondents (51.6%) were male, and 5.4% identified as LGB. Compared with heterosexual students, a greater proportion of LGB students reported experiencing mistreatment in all categories, including humiliation (27.0% LGB students vs 20.7% heterosexual students; *P* < .001), mistreatment not specific to identity (17.0% vs 10.3%; *P* < .001), and mistreatment specific to gender (27.3% vs 17.9%; *P* < .001), race/ethnicity (11.9% vs 8.6%; *P* < .001), and sexual orientation (23.3% vs 1.0%; *P* < .001). Being LGB was associated with increased odds of burnout (adjusted odds ratio, 1.63 [95% CI, 1.41-1.89]); this association persisted but was attenuated after adjusting for mistreatment (odds ratio, 1.36 [95% CI, 1.16-1.60]). The odds of burnout increased in a dose-response manner with mistreatment intensity. Lesbian, gay, or bisexual students reporting higher mistreatment specific to sexual orientation had and 8-fold higher predicted probability of burnout compared with heterosexual students (19.8% [95% CI, 8.3%-31.4%] vs 2.3% [95% CI, 0.2%-4.5%]; *P* < .001). Mediation analysis showed that mistreatment accounts for 31% of the total association of LGB sexual orientation with overall burnout (*P* < .001).

**Conclusions and Relevance:**

This study suggests that LGB medical students are more likely than their heterosexual peers to experience burnout, an association that is partly mediated by mistreatment. Further work is needed to ensure that medical schools offer safe and inclusive learning environments for LGB medical students.

## Key Points

**Question:**

Is there an association between sexual orientation and burnout among US medical students, and is this association mediated by experiences of mistreatment?

**Findings:**

Cross-sectional surveys of 2016 and 2017 graduating US medical students showed significantly higher rates of perceived mistreatment and burnout symptoms among lesbian, gay, or bisexual (LGB) medical students compared with heterosexual students. Perceived experiences of mistreatment mediated the association between LGB sexual orientation and burnout but did not completely account for excess burnout reported by LGB students.

**Meaning:**

The findings of this study suggest that medical students in sexual minority groups report increased symptoms of burnout regardless of their perceived experiences of mistreatment.

## Teaser

This cross-sectional study evaluates whether medical student burnout differs by sexual orientation and whether this association is mediated by experiences of mistreatment.

## Introduction

Burnout is a long-term reaction to stress characterized by emotional exhaustion, depersonalization, cynicism, and feelings of decreased personal accomplishment. Among physicians, burnout has been associated with poor professionalism, decreased empathy, poor quality of care, increased risk of patient safety incidents, reduced patient satisfaction, physician attrition, and high economic costs.^1,2,3,4,5,6^ Nearly half of medical students report symptoms of burnout.^7^ Experiencing burnout as a medical student has been associated with thoughts of dropping out of medical school, unprofessional behavior, depressive symptoms, and suicidal ideation.^8,9,10,11,12,13,14^ Preventing and addressing burnout are essential to improve and maintain health care quality, physician professionalism, and a robust health care workforce.

Causes of burnout are multifactorial and include competing time demands, strained finances, and unattainable expectations of medical training.^9,11,14^ Mistreatment is a particularly important factor in the development of burnout.^15^ Surveys of medical students have shown not only an association between mistreatment and burnout but also a dose-response association between frequency of mistreatment and risk of burnout.^15^ Mistreatment and discrimination have been shown to be negatively associated with the medical education of trainees in minority racial/ethnic groups (ie, Hispanic/Latinx, non-Hispanic Black, and non-Hispanic Asian individuals); however, some studies have shown lower rates of burnout among physicians and medical trainees in minority racial/ethnic groups.^16,17^ Previous research has begun to investigate individual and institutional factors associated with medical student burnout broadly; however, to our knowledge, these factors have not been studied among lesbian, gay, or bisexual (LGB) medical students. Limited research has documented LGB physician experiences of mistreatment and discrimination.^18,19,20,21,22^ A higher proportion of LGB medical students report experiences of mistreatment and discrimination based on sexual orientation compared with heterosexual students.^22^ Lesbian, gay, or bisexual medical students have also reported concealing their sexual identity during medical school for fear of discrimination^23^ as well as increased depression, anxiety, and low self-rated health compared with heterosexual medical students.^24^ These findings suggest that LGB physicians and physicians in training may be subject to unique stressors that are associated with their ability to remain in the health care workforce.

To our knowledge, the association between sexual orientation and medical student burnout is currently unknown. This study describes the prevalence of burnout in a national, contemporary cohort of graduating medical students, examines differences by sexual orientation, and evaluates whether this association is mediated by perceived mistreatment. By better characterizing burnout among LGB medical students and how burnout may be mediated by perceived mistreatment, this study aims to inform efforts to improve the educational experience and retention of LGB physicians in training.

## Methods

We analyzed cross-sectional data from the 2016 and 2017 Association of American Medical Colleges (AAMC) medical school graduation questionnaire (GQ). The GQ is an annual national survey of graduating medical students that asks questions about preclinical, clinical, and elective medical education experiences. Students complete the GQ at the end of medical school, prior to graduation. Responses are confidential, and participation is voluntary. In 2016, the AAMC added questions to the GQ about sexual orientation and gender identity. Graduation questionnaire items included in the analysis are listed in the eAppendix in the Supplement. We conducted study analyses according to the Strengthening the Reporting of Observational Studies in Epidemiology (STROBE) reporting guideline.^25^ This study analyzes data from an already existing AAMC database; no separate data collection was conducted and no participant consent was obtained for our analysis, although respondents do consent to taking the survey. Participant consent for data analysis was waived as AAMC deidentified all data made available to the research team. As this study analyzed deidentified, retrospective data, the study was deemed exempt by the Yale University Institutional Review Board.

The primary study outcome, burnout, was measured using the Oldenburg Burnout Inventory for Medical Students, which has been adapted from the validated Oldenburg Inventory and used by the AAMC, medical educators, and researchers to assesses the severity of burnout among medical students in 2 dimensions: exhaustion and disengagement.^26,27,28,29^ There are 8 question items in each dimension, with responses coded on a scale of 0 (strongly disagree) to 3 (strongly agree). Scoring responses were transformed such that higher scores indicated increased burnout. There is no set cutoff that has been validated to indicate definitive burnout. To identify students with the highest self-reported burnout symptoms compared with their peers, we summed the response scores, creating a range of 0 to 24 for each burnout dimension, and made a dichotomous variable to capture the top quartile for each burnout dimension (exhaustion score ≥13; disengagement score ≥12). Our primary burnout outcome was defined as having a score in the top quartile for both the exhaustion and disengagement dimensions.

Response options for sexual orientation included bisexual, gay or lesbian, and heterosexual or straight. Given the small number of students who identified as a sexual minority, we combined bisexual and gay or lesbian into a single LGB category. Information about gender identity was not made available to the research team by the AAMC for analysis to reduce identifiability given the low response rates of students identifying as transgender or genderqueer.

We considered questions about negative personal experiences with faculty, residents, nurses, staff, and peers, such as public humiliation, being threatened, being harmed, or receiving unwanted sexual advances as well as negative experiences associated with one’s gender (survey specified gender, not sex), race/ethnicity, or sexual orientation, to constitute different forms of mistreatment. Not all forms of mistreatment are equivalent in their severity or potential individual effect. Taking this fact into account, as well as our specific focus on sexual orientation, we combined questions about negative experiences into the following mistreatment groups: humiliation, mistreatment not specific to identity (ie, being threatened, being physically harmed, or receiving unwanted sexual advances), mistreatment specific to race/ethnicity, mistreatment specific to gender, and mistreatment specific to sexual orientation (eAppendix in the Supplement).

Mistreatment question responses were on a 4-point scale of never, once, occasionally, or frequently. Responses reflect the frequency, not severity, of perceived mistreatment, so to quantify mistreatment, we combined responses within each of the aforementioned mistreatment question categories on an ordinal scale of never (no mistreatment experienced), single (1 form of mistreatment experienced once), moderate (2 forms of mistreatment experienced once or 1 or 2 forms of mistreatment experienced occasionally), and high (≥3 forms of mistreatment experienced once or occasionally or any form of mistreatment experienced frequently).

Demographic characteristics included age at graduation, sex, race/ethnicity, and marital status. Other individual characteristics included receipt of scholarship and school loans. Students self-identified their race/ethnicity in multiple categories. For the regression analysis, students were categorized into 1 of 4 race/ethnicity groups: White, underrepresented in medicine (URM), not-White and not-URM, and other/unknown. We defined racial and ethnic identities as being underrepresented in medicine for racial and ethnic identities that are disproportionately underrepresented in the medical workforce relative to the general US population and include people who identify as American Indian, Alaska Native, Native Hawaiian, or other Pacific Islander; Black or African American; Hispanic, Latino, or of Spanish origin; or multiracial with 1 identity being an URM race and/or ethnicity. Students who were categorized as not-White and not-URM identified as Asian or multiracial with 1 identity being Asian and other identities not being URM. Students with other/unknown race/ethnicity either did not respond to the race/ethnicity questions or identified with a race/ethnicity not listed as a response option. Medical school characteristics included private or public status and mean institutional burnout score.

Respondents were excluded if they did not respond to the sexual orientation, mistreatment, or burnout questions (14.8% respondents excluded [eFigure 1 in the Supplement]). To assess for bias due to missing data, we compared demographic characteristics, reported mistreatment, and reported burnout for the full sample (n = 30 651) vs the analytic sample (n = 26 123). Excluded respondents were slightly older and tended to be Black or African American, American Indian, Alaska Native, Native Hawaiian, or other Pacific Islander but were not otherwise different in terms of sex, sexual orientation, marital status, school type, receipt of scholarship, or having loans. There were no differences in reported mistreatment and reported burnout between excluded respondents and the analytic sample.

### Statistical Analysis

Statistical analyses were performed from March 15, 2019, to July 2, 2020, and from November 20 to December 9, 2020. We used χ^2^ tests to compare covariates by sexual orientation and to assess the associations of sexual orientation with mistreatment and burnout. We constructed logistic regression models to evaluate the association between sexual orientation and burnout after adjusting for student demographic characteristics and medical school characteristics. To reduce identifiability, the AAMC did not provide an institution-level identifier for analysis. Instead, the AAMC calculated a categorical variable for mean institutional burnout divided into 6 categories (≤18, 19, 20, 21, 22, or 23; a higher score indicates a higher level of burnout). Therefore, to account for clustering by institution, we used a mixed-effects model to fit a random intercept for AAMC-calculated mean institutional burnout.

We used an iterative regression modeling approach to assess the association between sexual orientation and burnout and whether this association differed by experiences of mistreatment. To do this, we added categories of mistreatment in a stepwise manner, first including mistreatment specific to sexual orientation, then humiliation, mistreatment not specific to identity, mistreatment specific to race/ethnicity, and mistreatment specific to gender. We included interaction terms for sexual orientation and mistreatment specific to sexual orientation, race/ethnicity and mistreatment specific to race/ethnicity, and sex and mistreatment specific to gender to account for possible effect modification. To assess the degree to which mistreatment mediates the association between burnout and sexual orientation, we used the Stata module ldecomp (StataCorp LLC)^30,31^ to estimate the mean mediation effect of mistreatment specific to sexual orientation on the association between sexual orientation and burnout. Ldecomp allows for use of multiple mediators of any distribution and a binary outcome, such as burnout.^30,31,32^ Statistical significance was defined as a 2-sided *P* < .05. We used Stata, version 15 (StataCorp LLC) for all analyses.

## Results

In 2016 and 2017, there were 30 651 unique responses to the GQ from 38 160 eligible students (80.3% response rate) from 140 AAMC-accredited medical schools. After removal of incomplete responses, the final analytic sample contained 26 123 responses consisting of 68.5% of medical students at allopathic US medical schools in 2016 and 2017 (eFigure 1 in the Supplement). Most respondents (82.9%) were younger than 30 years and White (60.3%) (Table 1). A total of 12 653 respondents (48.4%) were female, 13 470 (51.6%) were male, and a minority (5.4%) identified as LGB. More than half the respondents received financial scholarship assistance (62.8%) or loans (73.2%) (Table 1).

**Table 1.** Characteristics of Graduating US Medical Students, by Sexual Orientation, 2016-2017

Characteristic	Medical students, No. (%)		
	Total	Heterosexual	LGB
Total	26 123 (100)	24 713 (94.6)	1410 (5.4)
Age, y			
≤26	10 908 (41.8)	10 375 (42.0)	533 (37.8)
27-29	10 748 (41.1)	10 153 (41.1)	595 (42.2)
30-32	2979 (11.4)	2806 (11.4)	173 (12.3)
≥33	1488 (5.7)	1379 (5.6)	109 (7.7)
Sex			
Male	13 470 (51.6)	12 643 (51.2)	827 (58.7)
Female	12 653 (48.4)	12 070 (48.8)	583 (41.4)
Race/ethnicity			
White	15 740 (60.3)	14 846 (60.1)	894 (63.4)
URM^a^	3850 (14.7)	3619 (16.7)	231 (16.4)
American Indian, Alaska Native, Native Hawaiian, or Pacific Islander	74 (0.3)	71 (0.3)	3 (0.2)
Black or African American	1296 (5.0)	1248 (5.1)	48 (3.4)
Hispanic	901 (3.5)	829 (3.4)	72 (5.1)
Multiracial, URM	1579 (6.0)	1471 (6.0)	108 (7.7)
Not Whie, not URM	6032 (23.1)	5756 (23.3)	276 (19.6)
Asian	5355 (20.5)	5130 (20.8)	225 (16.0)
Multiracial, not URM	677 (2.6)	626 (2.5)	51 (3.6)
Other^b^	253 (1.0)	249 (1.0)	4 (0.3)
Unknown	248 (1.0)	243 (1.0)	5 (0.4)
Marital status			
Single	19 121 (73.2)	17 935 (72.6)	1186 (84.1)
Married, common law, or civil union	6672 (25.5)	6476 (26.2)	196 (13.9)
Divorced, separated, or widowed	300 (1.2)	273 (1.1)	27 (1.9)
Missing	30 (0.1)	29 (0.1)	1 (0.1)
Type of medical school			
Public	10 247 (39.2)	9613 (38.9)	634 (45.0)
Private	15 876 (60.8)	15 100 (61.1)	776 (55.0)
Received scholarship			
Yes	16 407 (62.8)	15 486 (62.7)	921 (65.3)
No	9707 (37.2)	9220 (37.3)	487 (34.5)
Missing	9 (0.03)	7 (0.03)	2 (0.1)
Have medical school loans			
Yes	19 125 (73.2)	18 043 (73.0)	1082 (76.7)
No	6951 (26.6)	6625 (26.8)	326 (23.1)
Missing	47 (0.2)	45 (0.2)	2 (0.1)

Abbreviations: LGB, lesbian, gay, or bisexual; URM, underrepresented in medicine.

^a^ Defined as medical students who identified their race or ethnicity as underrepresented in medicine and includes people who identify as American Indian, Alaska Native, Native Hawaiian, or other Pacific Islander; Black or African American; Hispanic, Latino, or of Spanish origin; or multiracial with 1 identity being an URM race and/or ethnicity.

^b^ “Other” is a self-selected designation that indicates the race/ethnicity is not listed.

Lesbian, gay, or bisexual medical students had a disproportionately higher proportion of burnout compared with heterosexual medical students (17.2% LGB vs 11.1% heterosexual students; *P* < .001) (Table 2). This disproportionate difference was also true for the disengagement subscale (27.8% LGB vs 21.0% heterosexual students; *P* < .001) and exhaustion subscale (30.6% LGB vs 22.5% heterosexual students; *P* < .001).

**Table 2.** Graduating US Medical Student Burnout, by Sexual Orientation, 2016-2017^a^

Characteristic	Medical students, No. (%)			*P* value
	Total (N = 26 123)	Heterosexual (n = 24 713)	LGB (n = 1410)	
Burnout (upper quartile)	2997 (11.5)	2754 (11.1)	243 (17.2)	<.001
Disengagement (upper quartile)	5580 (21.4)	5188 (21.0)	392 (27.8)	<.001
Exhaustion (upper quartile)	6002 (23.0)	5571 (22.5)	431 (30.6)	<.001

Abbreviation: LGB, lesbian, gay, or bisexual.

^a^ Proportion of students with burnout and upper quartile scores for disengagement and exhaustion. Burnout is defined as having scores in the upper quartile for both disengagement and exhaustion dimensions of the Oldenburg Burnout Inventory for Medical Students.

One in 5 medical students (21.0%) reported experiencing some type of perceived public humiliation, 1 in 10 (10.6%) experienced perceived mistreatment not specific to identity, 8.7% experienced mistreatment specific to race/ethnicity, 18.4% experienced mistreatment specific to gender and a minority (2.2%) reported perceived mistreatment specific to their sexual orientation (Table 3). Lesbian, gay, or bisexual students reported a higher frequency of perceived mistreatment in all categories. More than one-fourth of LGB students (27.0%) reported being publicly humiliated compared with 1 in 5 (20.7%) heterosexual students (*P* < .001). A larger proportion of LGB students reported any perceived experience of mistreatment not specific to identity compared with heterosexual students (17.0% LGB vs 10.3% heterosexual students; *P* < .001). More than one-fourth (27.3%) of LGB students reported perceived mistreatment specific to gender compared with 17.9% of heterosexual students (*P* < .001). A total of 11.9% of LGB students reported perceived mistreatment specific to race/ethnicity compared with 8.6% of heterosexual students (*P* < .001). More than 1 in 5 (23.3%) LGB medical students reported perceived mistreatment specific to their sexual orientation at least once during medical school compared with 1.0% of heterosexual students, and LGB students reported moderate or high perceived mistreatment associated with their sexual orientation far more frequently than heterosexual students (12.6% vs 0.6%; *P* < .001).

**Table 3.** Graduating US Medical Student Experiences of Mistreatment, by Sexual Orientation, 2016-2017^a^

Experience	Medical students, No. (%)			*P* value
	Total (N = 26 123)	Heterosexual (n = 24 713)	LGB (n = 1410)	
Humiliation				
Never	20 626 (79.0)	19 596 (79.3)	1030 (73.0)	<.001
Single	3270 (12.5)	3072 (12.4)	198 (14.0)	
Moderate	2097 (8.0)	1934 (7.8)	163 (11.6)	
High	130 (0.5)	111 (0.4)	19 (1.3)	
Mistreatment not specific to identity				
Never	23 342 (89.4)	22 172 (89.7)	1170 (83.0)	<.001
Single	1756 (6.7)	1621 (6.6)	135 (9.6)	
Moderate	874 (3.3)	784 (3.2)	90 (6.4)	
High	151 (0.6)	136 (0.6)	15 (1.1)	
Mistreatment specific to gender				
Never	21 310 (81.6)	20 285 (82.1)	1025 (72.7)	<.001
Single	2093 (8.0)	1958 (7.9)	135 (9.6)	
Moderate	2182 (8.4)	1988 (8.0)	194 (13.8)	
High	538 (2.1)	482 (2.0)	56 (4.0)	
Mistreatment specific to race/ethnicity				
Never	23 839 (91.3)	22 597 (91.4)	1242 (88.1)	<.001
Single	995 (3.8)	933 (3.8)	62 (4.4)	
Moderate	904 (3.5)	824 (3.3)	80 (5.7)	
High	385 (1.5)	359 (1.5)	26 (1.8)	
Mistreatment specific to sexual orientation				
Never	25 544 (97.8)	24 463 (99.0)	1081 (76.7)	<.001
Single	253 (1.0)	102 (0.4)	151 (10.7)	
Moderate	239 (0.9)	96 (0.4)	143 (10.1)	
High	87 (0.3)	52 (0.2)	35 (2.5)	

Abbreviation: LGB, lesbian, gay, or bisexual.

^a^ Graduating US medical student experiences of mistreatment grouped into categories of humiliation, mistreatment not related to sexual orientation, and sexual orientation–related mistreatment by sexual orientation. Corresponding questions in each category are in the eAppendix in the Supplement. Never indicates no mistreatment; single, 1 form of mistreatment once; moderate, 1 or 2 forms of mistreatment occasionally or 2 forms once; and high, 3 or more forms of mistreatment once or occasionally or any form frequently.

In the multivariable model, being LGB was associated with significantly higher odds of burnout after adjusting for student demographic and medical school characteristics (odds ratio [OR], 1.63 [95% CI, 1.41-1.89]) (Table 4). After adjustment for all forms of perceived mistreatment, the association between identifying as LGB and burnout was attenuated but persisted (OR, 1.36 [95% CI, 1.16-1.60]). Being LGB remained significantly associated with burnout after including interactions between mistreatment specific to race and race/ethnicity and mistreatment specific to gender and sex. There was a significant interaction between mistreatment specific to race and race/ethnicity for overall burnout but not for the burnout or disengagement subscales. Although there was no interaction between mistreatment specific to gender and sex for overall burnout, there was a significant interaction between mistreatment specific to gender and sex for the disengagement burnout dimension. Results from the mediation analysis showed that perceived mistreatment accounts for 31% of the total association of LGB sexual orientation with burnout (*P* < .001).

**Table 4.** Graduating US Medical Student Sexual Orientation, Mistreatment, and Odds of Burnout, 2016-2017^a^

Characteristic	Adjusted for individual student demographic and medical school characteristics and mistreatment	
	OR (95% CI)	*P* value
Sexual orientation^b^		
Heterosexual	1 [Reference]	<.001
LGB	1.36 (1.16-1.60)	
Humiliation		
Never	1 [Reference]	<.001
Single	1.82 (1.63-2.03)	
Moderate	3.18 (2.82-3.57)	
High	5.51 (3.73-8.16)	
Mistreatment not specific to identity		
Never	1 [Reference]	<.001
Single	1.28 (1.12-1.47)	
Moderate	1.58 (1.33-1.88)	
High	1.12 (0.73-1.72)	
Mistreatment specific to gender		
Never	1 [Reference]	<.001
Single	1.45 (1.27-1.66)	
Moderate	1.58 (1.38-1.80)	
High	2.00 (1.60-2.53)	
Mistreatment specific to race/ethnicity		
Never	1 [Reference]	.12
Single	1.12 (0.93-1.34)	
Moderate	1.23 (1.03-1.48)	
High	1.03 (0.78-1.36)	
Mistreatment specific to sexual orientation		
Never	1 [Reference]	.08
Single	1.20 (0.86-1.67)	
Moderate	1.27 (0.92-1.75)	
High	0.59 (0.33-1.04)	

Abbreviations: LGB, lesbian, gay, or bisexual; OR, odds ratio.

^a^ Odds of burnout adjusted for demographic characteristics, mistreatment, and mean institutional burnout. Burnout is defined as having scores in the upper quartile for both disengagement and exhaustion dimensions of the Oldenburg Burnout Inventory for Medical Students. Demographic and medical school characteristics included in model: age, sex, race/ethnicity, marital status, type of medical school, school loans, and receipt of scholarship. Models included a random effect to account for clustering by institution, using a mixed-effects model to fit a random intercept for Association of American Medical Colleges–calculated mean institutional burnout provided in 1 of 6 categories (≤18, 19, 20, 21, 22, or 23). Never indicates no mistreatment; single, 1 form of mistreatment once; moderate, 1 or 2 forms of mistreatment occasionally or 2 forms once; and high, 3 or more forms of mistreatment once or occasionally or any form frequently.

^b^ The OR for LGB sexual orientation vs heterosexual, adjusted for individual student demographic and medical school characteristics, is 1.63 (95% CI, 1.41-1.89; *P* < .001).

There was a dose-response association between perceived mistreatment intensity and risk of burnout. After adjustment for student demographic and medical school characteristics, the odds of burnout among students who experienced a single episode of humiliation were significantly higher compared with individuals who experienced none (OR, 1.82 [95% CI, 1.63-2.03]) (Table 4). The odds of burnout were even higher for students who had moderate (OR, 3.18 [95% CI, 2.82-3.57]) or high (OR, 5.51 [95% CI, 3.73-8.16]) perceived experiences of humiliation. This dose-response association was also seen with increased frequency of perceived mistreatment specific to gender, with ORs (in comparison with students with no experiences of mistreatment) increasing in a stepwise manner after a single episode of mistreatment specific to gender (OR, 1.45 [95% CI, 1.27-1.66]) or moderate perceived mistreatment (OR, 1.58 [95% CI, 1.38-1.80]) and more than doubling for high perceived mistreatment specific to gender (OR, 2.00 [95% CI, 1.60-2.53]). There was no dose-response trend seen with mistreatment not specific to identity or mistreatment specific to race/ethnicity.

In the fully adjusted model, there was a significant interaction between perceived sexual orientation–specific mistreatment and LGB status. There was no difference in burnout between LGB and heterosexual students who did not perceive an experience of sexual orientation–specific mistreatment. However, the interaction between sexual orientation and sexual orientation–specific mistreatment demonstrated dose-response differences in burnout between LGB and heterosexual students with increasing perceived sexual orientation–specific mistreatment (Figure). No difference in burnout was observed between LGB and heterosexual students with low perceived sexual orientation–specific mistreatment (Figure), but LGB students who experienced a high frequency of perceived sexual orientation–specific mistreatment were more than 8 times more likely to have burnout (predicted probability of high burnout score, 19.8% [95% CI, 8.3%-31.4%]) compared with heterosexual students (predicted probability of high burnout score, 2.3% [95% CI, 0.2%-4.5%]) (Figure). Similar trends were also observed for disengagement (eTable 1 and eFigure 2 in the Supplement) and exhaustion (eTable 2 and eFigure 3 in the Supplement).

**Figure.**
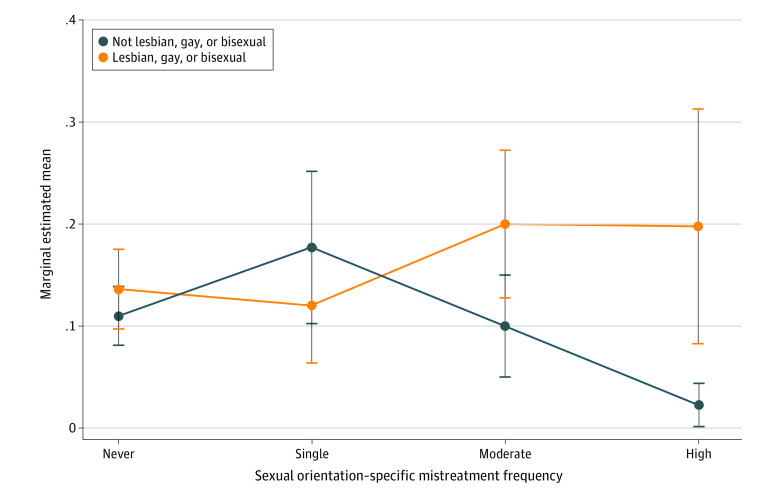
Association Between Graduating Medical Student Sexual Orientation–Specific Mistreatment and Burnout According to Sexual Orientation, 2016-2017 Burnout predictive margins with 95% CI of sexual orientation and frequency of sexual orientation–related mistreatment interaction (*P* < .001).

## Discussion

Medical students who identify as sexual minorities were much more likely to report perceived mistreatment of all types and had a greater likelihood of burnout compared with heterosexual students. Odds of having burnout increased in a dose-response manner with more frequent experiences of perceived mistreatment. Although perceived mistreatment is an important mediator of developing symptoms of burnout, it does not completely explain the excess burnout experienced by LGB students. Lesbian, gay, or bisexual medical students had a higher likelihood of all forms of mistreatment compared with their heterosexual peers and were more than 30% more likely to experience burnout even after adjusting for perceived mistreatment.

Our findings have important implications for creating healthy, diverse, and inclusive medical school learning environments and for the development and maintenance of an LGB workforce. The positive association between increasing intensity of perceived mistreatment and the probability of burnout is consistent with prior studies.^15^ Although we found that perceived mistreatment contributed to the association between being LGB and burnout symptoms, the association between perceived mistreatment and burnout differed across student groups; LGB students experiencing high perceived sexual orientation–specific mistreatment had more than 8 times higher probability of burnout compared with heterosexual students.^33^ Significantly higher odds of burnout were also observed for students experiencing mistreatment specific to gender.

Mistreatment is just 1 symptom of a hostile learning environment. Factors that may create a hostile learning environment for LGB trainees include microaggressions, fear of discrimination, overt homophobia and discrimination, and internalized stigma.^34,35,36,37,38^ Prior studies have demonstrated that LGB medical students experience higher levels of mistreatment and discrimination compared with heterosexual students^22^ and conceal their sexual orientation for fear of discrimination or mistreatment.^23^ Although this study does not measure whether someone is “out” (that they are public about their sexual orientation), the mental health consequences of LGB minority stress—chronically elevated stress as a result of discrimination and prejudice directed toward sexual and gender minorities^39^—are well described^24,25,26,27,28^ and include increased rates of depression, anxiety, suicidal ideation, and suicide attempts.^34,40,41^ Students are affected by both larger societal forces and medical school culture. Our study indicates that, for medical trainees, the minority stress of being an LGB physician in training may result in higher symptoms of burnout and should spur medical schools and clinical training sites to examine their culture and practices to create a training environment that is safe for and inclusive of sexual and gender minority groups. Further investigations are needed to more clearly understand the characteristics of minority stress due to being LGB in medical school and the effect it has on LGB trainees and physician retention, as well as interventions that can successfully reduce medical student mistreatment, burnout, and minority stress.

Burnout has complex underpinnings, and its causes are multifactorial.^42,43,44^ Successfully preventing and addressing medical student burnout will require identifying the causes of burnout and addressing many different factors. As we demonstrated, mistreatment does not fully account for the association between being a sexual minority and burnout. Addressing both mistreatment and minority stress associated with sexual orientation will require a comprehensive assessment of medical school culture, environment, and curricula.

Creating a welcoming medical school environment where lesbian, gay, bisexual, transgender, queer, and intersex (LGBTQI+) students can thrive will require many institutional changes. Some medical schools have implemented programs and policies beyond cultural competency training to create a positive environment for LGBTQI+ students.^45^ This environment includes financial support for LGBTQI+ student and faculty organizations, an organized presence of out and visible sexual and gender minority faculty and allies who can provide support and mentorship to LGBTQI+ trainees, LGBTQI+ health electives, and accurate and meaningful inclusion of sex, gender, sexual orientation, and related patient care topics in medical school curricula.^46,47,48^ Although beyond the scope of this analysis, in addition to having well-trained and supportive faculty and staff, other institutional measures to ensure that LGBTQI+ students feel safe and supported include the provision of LGBTQI+ health benefits, which can have a significant association with student well-being.^49^ This support includes benefit coverage for sexual minority couples, gender-affirming medications and procedures, and preexposure prophylaxis for all students regardless of sexual orientation.^49^

### Limitations

This study has several limitations. First, all data are by self-report and may be subject to response and recall bias. Second, the cross-sectional nature of this study can identify only associations, not causal relationships. Third, gender identity and nonbinary sex are not included in our study model, so we are unable to account for perceived mistreatment and/or burnout among students who are intersex, transgender, gender nonbinary, or gender nonconforming. This factor is particularly important given the significantly higher odds of burnout observed for students experiencing mistreatment based on gender. Fourth, excluded respondents were slightly older, and a higher proportion were Black or African American, American Indian, Alaska Native, Native Hawaiian, or other Pacific Islander. Although these differences were not large, they may affect the generalizability of our findings to these groups of medical students. Fifth, this analysis does not account for the effect of multiple identities and differences in comfort identifying and reporting mistreatment among student subgroups, both of which may compound experiences of perceived mistreatment and burnout.

Several factors affect our assessment of sexual orientation, mistreatment, and burnout among medical students. First, fear of being identified as a sexual minority and limited sexual orientation response categories may result in underreporting of sexual minority medical students in our study sample. Current survey response categories for sexual orientation exclude students who do not identify as heterosexual, lesbian, gay, or bisexual. This categorization would exclude people with other sexual identities, such as individuals who identify as queer or asexual. Furthermore, the true prevalence of LGB status among medical students is not known, so we are unable to accurately estimate response bias that may be associated with study outcomes. Perceived mistreatment may also be underreported owing to fear of identification and the possible consequences if a student’s identity was linked to responses. Mistreatment and burnout are also not evenly distributed across all institutions. To account for differential levels of burnout across medical schools, we included mean institutional burnout scores in our regression models, which did not attenuate the association between LGB sexual orientation and burnout. More important, this study also does not specify the source of mistreatment, whether from faculty, peers, or hospital staff. Although training and policies are needed across all medical schools and health care sectors to eliminate mistreatment and address homophobia, knowing where and from whom students are experiencing mistreatment could guide future interventions to create a learning environment that is safe for and inclusive of individuals in sexual and gender minority groups. Finally, the GQ is administered near the completion of the fourth and final year of medical school and after the residency match. Therefore, survey responses may be either attenuated or accentuated by recall bias and may not reflect perceptions and experiences of medical students earlier in their training.

## Conclusions

In this study, lesbian, gay, or bisexual medical students reported higher levels of burnout and mistreatment compared with their heterosexual peers. To build a healthy and high-performing LGB physician workforce, systemic, multidimensional approaches are needed to reduce both mistreatment and minority stress experienced by sexual minority medical students.

## Notes

Supplement. **eAppendix.** Questions Included for Analysis From the 2016 and 2017 AAMC Medical School Graduation Questionnaire **eFigure 1.** Participant Study Flow Diagram **eFigure 2.** Relation Between Graduating Medical Student Sexual Orientation–Specific Mistreatment and Burnout Due to Disengagement According to Sexual Orientation, 2016-2017 **eFigure 3.** Relation Between Graduating Medical Student Sexual Orientation–Specific Mistreatment and Burnout Due to Exhaustion According to Sexual Orientation, 2016-2017 **eTable 1.** Graduating U.S. Medical Student Sexual Orientation, Mistreatment, and Odds of Being in the Highest Disengagement Burnout Quartile, 2016-2017 **eTable 2.** Graduating U.S. Medical Student Sexual Orientation, Mistreatment, and Odds of Being in the Highest Exhaustion Burnout Quartile, 2016-2017
