# Supplementary material for: Association Between Sexual Orientation, Mistreatment, and Burnout Among US Medical Students
Source: JAMA Netw Open. 2021 Feb 2;4(2):e2036136. doi: 10.1001/jamanetworkopen.2020.36136 (PMC7856540; doi:10.1001/jamanetworkopen.2020.36136)
Supplement: Supplement. — eAppendix. Questions Included for Analysis From the 2016 and 2017 AAMC Medical School Graduation Questionnaire eFigure 1. Participant Study Flow Diagram eFigure 2. Relation Between Graduating Medical Student Sexual Orientation–Specific Mistreatment and Burnout Due to Disengagement According to Sexual Orientation, 2016-2017 eFigure 3. Relation Between Graduating Medical Student Sexual Orientation–Specific Mistreatment and Burnout Due to Exhaustion According to Sexual Orientation, 2016-2017 eTable 1. Graduating U.S. Medical Student Sexual Orientation, Mistreatment, and Odds of Being in the Highest Disengagement Burnout Quartile, 2016-2017 eTable 2. Graduating U.S. Medical Student Sexual Orientation, Mistreatment, and Odds of Being in the Highest Exhaustion Burnout Quartile, 2016-2017 [file jamanetwopen-e2036136-s001.pdf]

Supplementary Online Content

Samuels EA, Boatright DH, Wong AH, et al. Association between sexual orientation, mistreatment, and burnout among US medical students. *JAMA Netw Open.* 2021;4(2):e2036136. doi:10.1001/jamanetworkopen.2020.36136

eAppendix. Questions Included for Analysis From the 2016 and 2017 AAMC Medical School Graduation Questionnaire
eFigure 1. Participant Study Flow Diagram
eFigure 2. Relation Between Graduating Medical Student Sexual Orientation–Specific Mistreatment and Burnout Due to Disengagement According to Sexual Orientation, 2016-2017
eFigure 3. Relation Between Graduating Medical Student Sexual Orientation–Specific Mistreatment and Burnout Due to Exhaustion According to Sexual Orientation, 2016-2017
eTable 1. Graduating U.S. Medical Student Sexual Orientation, Mistreatment, and Odds of Being in the Highest Disengagement Burnout Quartile, 2016-2017
eTable 2. Graduating U.S. Medical Student Sexual Orientation, Mistreatment, and Odds of Being in the Highest Exhaustion Burnout Quartile, 2016-2017

This supplementary material has been provided by the authors to give readers additional information about their work.

© 2021 Samuels EA et al. *JAMA Network Open.*

eAppendix. Questions included for analysis from the 2016 and 2017 AAMC Medical School Graduation Questionnaire

Excerpt of questions from the AAMC Graduation Questionnaire included in the study analysis. Questions shown and numbered in order of appearance. Questions not included in analysis are omitted.

1. **Sex:**
   - Male
   - Female

2. **Age at graduation:**
   - Under 24
   - 24 through 26
   - 27 through 29
   - 30 through 32
   - Over 32

3. **How do you self-identify?**
   - American Indian or Alaska Native
   - Asian
   - Black or African American
   - Hispanic, Latino, or of Spanish origin
   - Native Hawaiian or Other Pacific Islander
   - White
   - Other

4. **What is your current marital status?**
   - Single (never legally married)
   - Legally married
   - Common law or civil union
   - Divorced
   - Separated, but still legally married
   - Widowed

23. Please indicate the extent to which you agree with the following statements:

	Strongly Agree	Disagree	Agree	Strongly Agree
I always find new and interesting aspects in my medical school work.				
There are days when I feel tired before I arrive at medical school.				
It happens more and more often that I talk about my medical school work in a negative way.				
After a day of medical school, I tend to need more time than in the past in order to relax and feel better.				
I can tolerate the pressure of my				

medical school work very well.				
Lately, I tend to think less at medical school and do my medical school work almost mechanically.				
I find my medical school work to be a positive challenge.				
During my medical school work, I often feel emotionally drained.				
Over time, one can become disconnected from medical school work.				
After a day of medical school, I have enough energy for my leisure activities.				
Sometimes I feel sickened by my medical school work.				
After a day of medical school, I usually feel worn out and weary.				
The study of medicine is the only thing that I can imagine myself doing.				
Usually, I can manage the amount of my medical school work well.				
I feel more and more engaged in my medical school work.				
When I am at medical school, I usually feel energized.				

39. For each of the following behaviors, please indicate the frequency you personally experienced that behavior during medical school. Include in your response any behaviors performed by faculty, nurses, residents/interns, other institution employees or staff, and other students. Please do not include behaviors performed by patients.

	Never	Once	Occasionally	Frequently
**Humiliation***				
Been publicly humiliated?				
**Mistreatment not specific to identity***				
Been threatened with physical harm?				
Been physically harmed?				
Been required to perform personal services?				
Been subjected to unwanted sexual advances?				
Been asked to exchange sexual favors for grades or other rewards?				
**Mistreatment specific to gender***				
Been denied opportunities for training or rewards based on gender?				

© 2021 Samuels EA et al. *JAMA Network Open.*

Been subjected to offensive sexist remarks/names?				
Received lower evaluations or grades solely because of gender rather than performance?				
**Mistreatment specific to race/ethnicity***				
Been denied opportunities for training or rewards based on race or ethnicity?				
Been subjected to racially or ethnically offensive remarks/names?				
Received lower evaluations or grades solely because of race or ethnicity rather than performance?				
**Mistreatment specific to sexual orientation***				
Been denied opportunities for training or rewards based on sexual orientation?				
Been subjected to offensive remarks/names related to sexual orientation?				
Received lower evaluations or grades solely because of sexual orientation rather than performance?				

Categories of humiliation, mistreatment not specific to identity, mistreatment specific to gender, mistreatment specific to race/ethnicity, and mistreatment specific to sexual orientation are not named in the original survey instrument. These titles are included here to indicate which questions correspond with these categories used in study analysis.

49. Did you receive any scholarships, stipends, or grants (not loans) for medical school?
- Yes
- No

53. Do you have any outstanding educational loans (including loan service commitments) for your medical school education?
- Yes
- No

62. How do you self-identify?
- Bisexual
- Gay or lesbian
- Heterosexual or straight

64. Control of medical school:
- Private
- Public

eFigure 1. Participant Study Flow Diagram

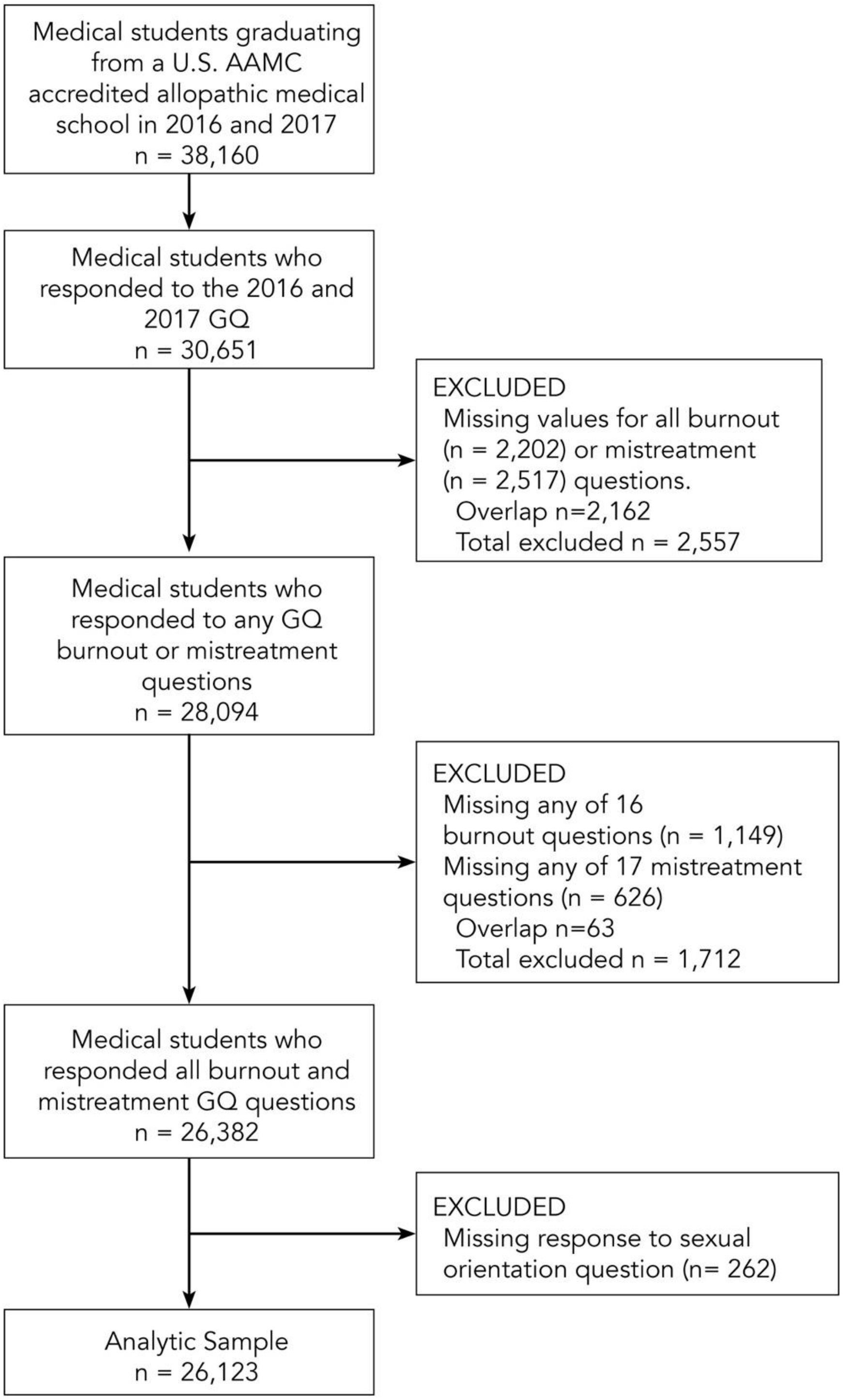

Supplemental Figure 1: Exclusion and inclusion of responses to the 2016 and 2017 AAMC Graduation Questionnaire. AAMC = Association of American Medical Colleges. GQ= Graduation Questionnaire

© 2021 Samuels EA et al. *JAMA Network Open.*

eTable 1. Graduating U.S. Medical Student Sexual Orientation, Mistreatment, and Odds of Being in the Highest Disengagement Burnout Quartile, 2016-2017

	Adjusted for individual demographics and medical school characteristics		Adjusted for individual demographics, medical school characteristics, and mistreatment	
	OR (95% CI)	*p*	OR (95% CI)	*p*
**Sexual Orientation**		<.001		<.001
Heterosexual	1		1	
LGB	1.39 [1.23,1.57]		1.21 [1.06,1.39]	
**Humiliation***				<.001
Never			1	
Single			1.53 [1.40, 1.67]	
Moderate			2.45 [2.21, 2.71]	
High			5.21 [3.48, 7.77]	
**Mistreatment not specific to identity***				<.001
Never			1	
Single			1.22 [1.09,1.37]	
Moderate			1.50 [1.29, 1.75]	
High			0.92 [0.61,1.38]	
**Mistreatment specific to gender***				<.001
Never			1	
Single			1.34 [1.20, 1.50]	
Moderate			1.57 [1.40, 1.76]	
High			2.15 [1.74, 2.65]	
**Mistreatment specific to race***				.008
Never			1	
Single			1.11 [0.95, 1.29]	
Moderate			1.28 [1.09, 1.50]	
High			1.24 [0.97, 1.58]	
**Mistreatment specific to sexual orientation***				0.012
Never			1	
Single			1.16 [0.87,1.55]	
Moderate			1.12 [0.84,1.50]	
High			0.45 [0.26, .76]	

<u>Supplemental Table 1:</u> *Odds of being in the highest quartile of disengagement burnout adjusted for demographics, mistreatment, and average institutional burnout* Demographic variables and medical school characteristics included in model: Age, sex, race, marital status, type of medical school, school loans, and receipt of scholarship. Models included a random effect to account for clustering by institution, we used a mixed-effects model to fit a random intercept for AAMC calculated average institutional burnout provided in one of 6 categories: <=18, 19, 20, 21, 22, or 23. *Never indicates no mistreatment; single 1 form of mistreatment once; moderate 1 or 2 forms of mistreatment occasionally or 2 forms of mistreatment once; high, 3 or more forms of mistreatment once or occasionally or any form of mistreatment frequently

© 2021 Samuels EA et al. *JAMA Network Open.*

eFigure 2. Relation Between Graduating Medical Student Sexual Orientation–Specific Mistreatment and Burnout Due to Disengagement According to Sexual Orientation, 2016-2017

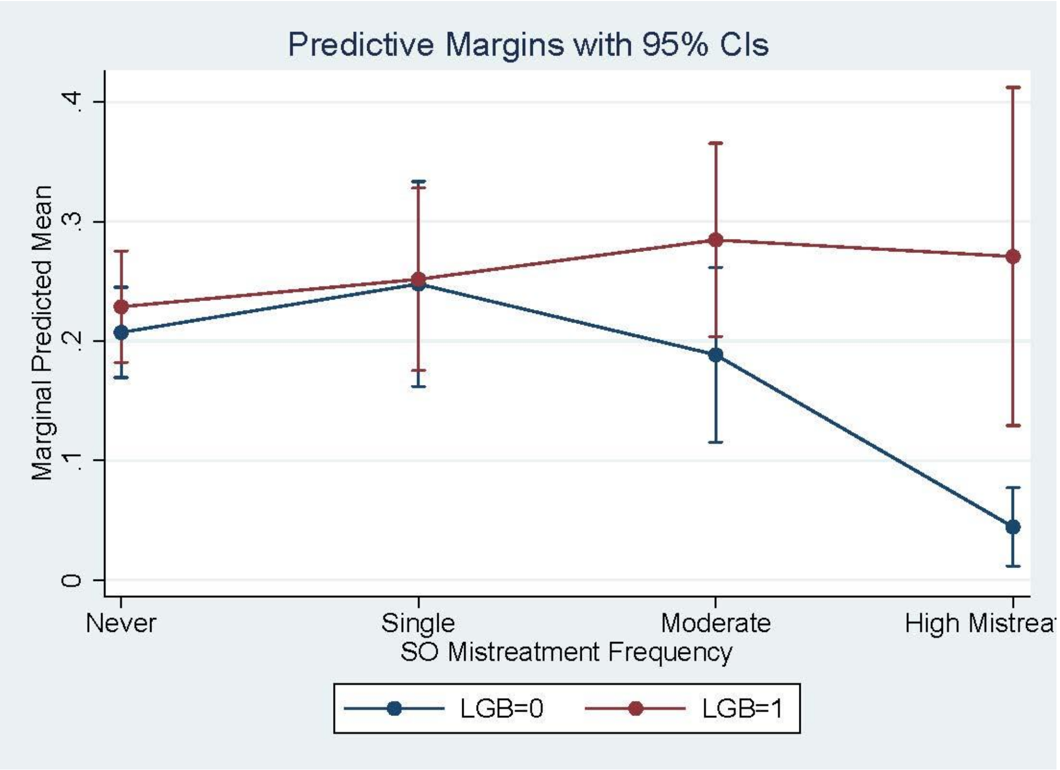

Supplemental Figure 2: Disengagement predictive margins with 95% CI of sexual orientation and frequency of mistreatment specific to sexual orientation interaction, p=.0006

© 2021 Samuels EA et al. *JAMA Network Open.*

**eTable 2.** Graduating U.S. Medical Student Sexual Orientation, Mistreatment, and Odds of Being in the Highest Exhaustion Burnout Quartile, 2016-2017				
	Adjusted for individual demographics and medical school characteristics		Adjusted for individual demographics, medical school characteristics, and mistreatment	
	OR (95% CI)	*p*	OR (95% CI)	*p*
**Sexual Orientation**		<.001		.002
Heterosexual	1		1	
LGB	1.53 [1.36,1.73]		1.33 [1.16,1.51]	
**Humiliation***				<.001
Never			1	
Single			1.78 [1.63, 1.93]	
Moderate			2.82 [2.54, 3.11]	
High			3.78 [2.56, 5.57]	
**Mistreatment not specific to identity***				<.001
Never			1	
Single			1.28 [1.15, 1.43]	
Moderate			1.44 [1.23, 1.67]	
High			1.05 [0.71, 1.55]	
**Mistreatment specific to gender***				<.001
Never			1	
Single			1.26 [1.14, 1.41]	
Moderate			1.42 [1.28, 1.58]	
High			1.69 [1.38, 2.08]	
**Mistreatment specific to race***				.008
Never			1	
Single			1.12 [0.97, 1.30]	
Moderate			1.28 [1.10, 1.49]	
High			1.16 [0.91, 1.48]	
**Mistreatment specific to sexual orientation***				0.572
Never			1	
Single			1.17 [0.88,1.55]	
Moderate			1.16 [0.87,1.55]	
High			0.957 [0.58, 1.59]	

<u>Supplemental Table 2:</u> *Odds of being in the highest quartile of exhaustion burnout adjusted for demographics, mistreatment, and average institutional burnout* Demographic variables and medical school characteristics included in model: Age, sex, race, marital status, type of medical school, school loans, and receipt of scholarship. Models included a random effect to account for clustering by institution, we used a mixed-effects model to fit a random intercept for AAMC calculated average institutional burnout provided in one of 6 categories: <=18, 19, 20, 21, 22, or 23. *Never indicates no mistreatment; single 1 form of mistreatment once; moderate 1 or 2 forms of mistreatment occasionally or 2 forms of mistreatment once; high, 3 or more forms of mistreatment once or occasionally or any form of mistreatment frequently

© 2021 Samuels EA et al. *JAMA Network Open.*

eFigure 3. Relation Between Graduating Medical Student Sexual Orientation–Specific Mistreatment and Burnout Due to Exhaustion According to Sexual Orientation, 2016-2017

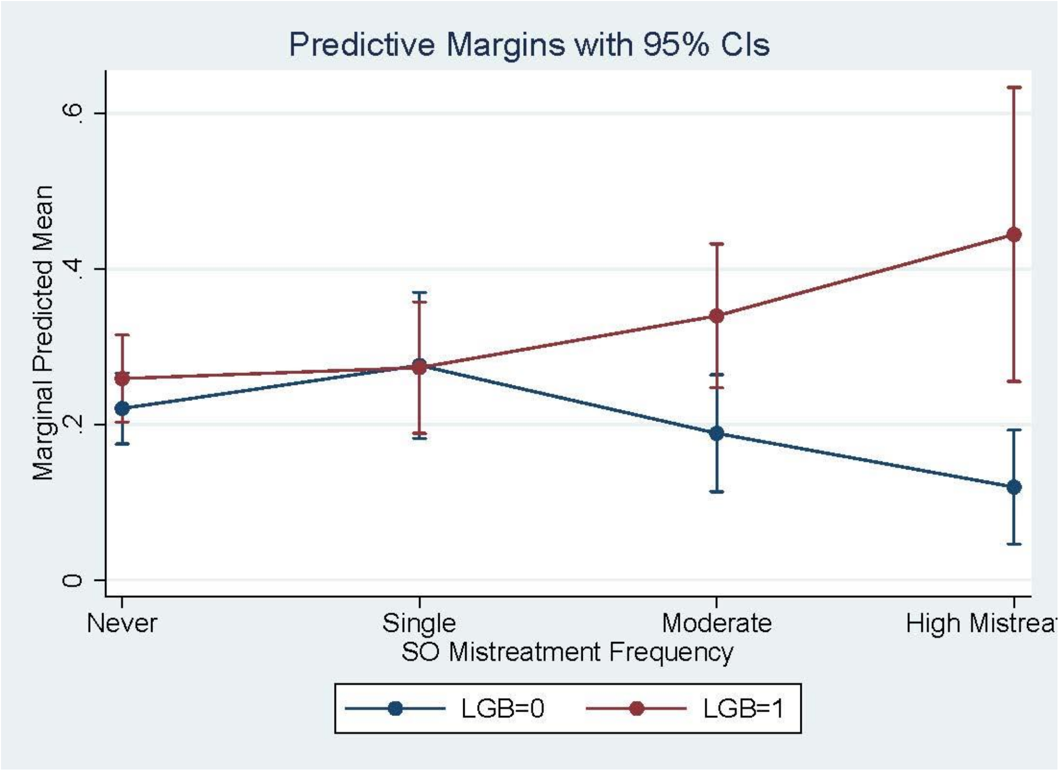

Supplemental Figure 3: Exhaustion predictive margins with 95% CI of sexual orientation and frequency of mistreatment specific to sexual orientation interaction, p=.0013

© 2021 Samuels EA et al. *JAMA Network Open.*
